# Flexible Stabilisation of the Degenerative Lumbar Spine Using PEEK Rods

**DOI:** 10.1155/2016/7369409

**Published:** 2016-02-15

**Authors:** Jacques Benezech, Bruno Garlenq, Gilles Larroque

**Affiliations:** ^1^Service de Neurochirurgie, Clinique du Millenaire, 34000 Montpellier, France; ^2^Service de Rééducation Fonctionnelle, Centre Hospitalier Paul Coste Floret, 34240 Lamalou les Bains, France; ^3^Invibio Ltd., Technology Centre, Hillhouse International, Thornton Cleveleys, Lancashire FY5 4QD, UK

## Abstract

Posterior lumbar interbody fusion using cages, titanium rods, and pedicle screws is considered today as the gold standard of surgical treatment of lumbar degenerative disease and has produced satisfying long-term fusion rates. However this rigid material could change the physiological distribution of load at the instrumental and adjacent segments, a main cause of implant failure and adjacent segment disease, responsible for a high rate of further surgery in the following years. More recently, semirigid instrumentation systems using rods made of polyetheretherketone (PEEK) have been introduced. This clinical study of 21 patients focuses on the clinical and radiological outcomes of patients with lumbar degenerative disease treated with Initial VEOS PEEK^®^-Optima system (Innov'Spine, France) composed of rods made from PEEK-OPTIMA^®^ polymer (Invibio Biomaterial Solutions, UK) without arthrodesis. With an average follow-up of 2 years and half, the chances of reoperation were significantly reduced (4.8%), quality of life was improved (ODI = 16%), and the adjacent disc was preserved in more than 70% of cases. Based on these results, combined with the biomechanical and clinical data already published, PEEK rods systems can be considered as a safe and effective alternative solution to rigid ones.

## 1. Introduction

**Figure 1:**
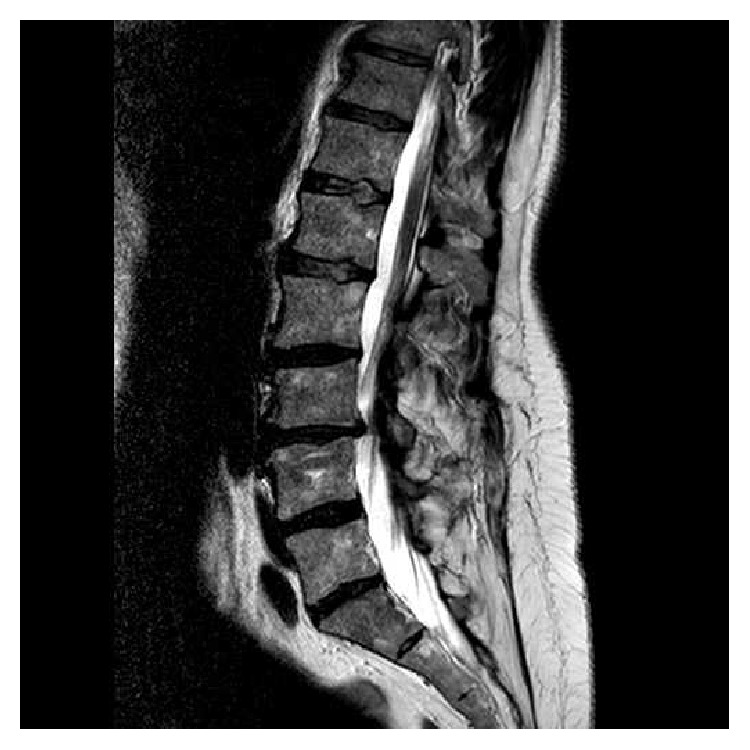
Sagittal T1-weighted MR imaging of the lumbar spine of a 55-yo patient with a previous rigid fixation on L4L5S1 level shows adjacent segment disease at the L3L4 level.

Symptomatic lumbar degenerative disease is essentially characterised by pains and walking difficulties due to abnormal motion or compression of neural structures and their vessels. This reflects specific situations, such as narrowing of the spinal canal, degenerative disc disease, and herniated disc, as well as any degenerative impairment of the posterior arch (arthropathy, spondylolisthesis, etc.). Most of the time surgical treatment is necessary to reduce the symptoms, with arthrodesis generally regarded as being the treatment of choice for this pathology [1, 2]. The use of pedicle screw instrumentation (comprising metal rods) ensures immediate stabilisation and increases the chances of fusion: this technique is therefore preferred, today, by a number of authors [3–7]. Nevertheless, in more than 40% of cases the persistence of symptoms, the progression of the degenerative disease, or the appearance of new symptoms related to the initial operation leads to a further operation [8]. This induced pathology is known as “adjacent segment disease” (ASD) (Figure 1), with a varying incidence that could reach 30% within 1 year and 100% within 10 years, with a rate of 35% to 45% being the most frequently noted [9–11]. Several authors raise the question of the rigidity of these constructs [12, 13] that would significantly increase stress on the discs and adjacent joints, leading to hypertrophy of these facets, the formation of osteophytes, hyper segmental motion, and lumbosacral stenosis [14–16]. Dynamic systems (nonfusion) have been proposed to reduce the risk of arthrodesis-induced complications. Nevertheless, no significant difference in terms of rate of complications and surgical revisions has so far been demonstrated between dynamic stabilisation and fusion [17].

PEEK (polyether ether ketone) is a synthetic polymer material fully biocompatible [18, 19], with a low elastic modulus, similar to that of bone (3.6 GPa) [20], and less rigid than titanium (115 GPa). PEEK is also known as a radiotransparent material, reducing artefacts during radiological investigations [21]. It has been used for spinal implants since 1990 [22, 23], first as an interbody cage and more recently for rods.

In this study, we are reporting a series of 21 patients treated with Initial VEOS PEEK-Optima (Innov'Spine, France) composed of rods made from PEEK-OPTIMA polymer (Invibio Biomaterial Solutions), without arthrodesis and with a minimum of 2.5-year follow-up. The following clinical outcomes were studied: early and late complications, reoperation, pain (VAS), Oswestry Disability Index (ODI), patients' satisfaction, and evolution of the adjacent disc spaces. Our objective was to validate this technique by demonstrating the values of PEEK-OPTIMA rods system compared to rigid one based on these parameters.

## 2. Materials and Method

This study has been approved by the National Authority Bodies: French National Commission for Information Technology and Civil Liberties (CNIL) and the Advisory Committee on Information Processing in Material Research in the Field of Health (CCTIRS) in order to comply with local ethical and regulatory requirements.

Out of a homogeneous cohort of 41 patients operated on between 1 June 2011 and 30 June 2012, all with advanced degenerative lumbar disease resistant to medical treatment, 21 were selected as meeting the criteria laid down for inclusion in the study:

1. patient with degenerative disease of the lumbar column resistant to medical treatments and over 21 years of age,
2. patient operated on with the Initial VEOS PEEK-Optima (Innov'Spine, France) system,
3. surgery performed after 2011,
4. patient agreeing to participate, provide personal health information (PHI), and sign the informed consent form.

Patients operated on with another osteosynthetic system, by anterior arthrodesis or by arthroplasty, were excluded.

All the patients were operated on by the same surgeon in accordance with the same protocol comprising a posterior approach, any treatment of a herniated disc, and the widening of the lumbar canal if necessary, as well as stabilisation of the relevant disc spaces with PEEK-OPTIMA rods and pedicle screws. Surgery was at only 1 level in 14 cases (i.e., 66.7% of the patients) and at 2 or more levels in 7 cases (i.e., 33.3% of the patients).

The surgical indications to choose this type of implants were canal stenosis (*N* = 9, 43%), degenerative disc diseases (DDD) (*N* = 2, 10%), canal stenosis + DDD (*N* = 2, 10%), canal stenosis + spondylolisthesis (*N* = 7, 32%), soft disc herniation (*N* = 1, 5%), and canal stenosis + soft disc herniation + spondylolisthesis (*N* = 1, 5%).

Twelve men (57%) and nine (43%) women were included. The average age of 70 years is in line with the typical degenerative aetiology of this segment of the population. Pain was mainly lumbar-radicular (*N* = 16, 76.2%). The most frequently reached level was L4L5 (*N* = 10, 47.6%).

The average duration of the follow-up was 29.3 months (std. 2.2) with a median of 29 months (IQR 27–31), time between the date of the surgery and the last visit where the patients answer the questionnaire.

The global success of the surgery for each patient is defined as follows: no adverse event directly related to the device and no reintervention, at each follow-up evaluation (each visit and patient questionnaire).

The complication rate was calculated from all the adverse events (minor or major) observed by the surgeon during the follow-up visit or reported by the patient on the questionnaire.

Quality of life was evaluated using the visual analogue scale (VAS) and Oswestry Disability Index (ODI). We chose ODI [24–26] as it seemed the most reliable for assessing the problem's functional impact on the patient's everyday life. These two methods of evaluation (VAS and ODI) together with a patient satisfaction index (PSI) were applied to the patients in the form of a questionnaire for final clinical evaluation.

The PSI is evaluated on a scale of (1) to (4):

1. I am quite satisfied with my operation.
2. My condition has not improved as much as I wanted but I would be prepared to undergo the same operation for the same outcome.
3. The operation has improved my condition, but I would not be prepared to undergo the same operation for the same outcome.
4. My condition is the same or even worse than before my operation.

As regards the radiological aspect, all the patients had a preoperative MRI scan where one could evaluate the state of disc degeneration (dehydration, protrusion, and hernia), the impact on the vertebral end plates (Modic), and the amount and extent of the stenosis. For the postoperative radiological assessment, as we had no late postsurgery MRI scan imaging available for all the patients, we were limited to measuring the disc height taken at the centre of the intervertebral disc space prior to surgery and at the time of the last assessment, as well as the upper and lower adjacent discs.

The statistical analysis consisted of chi-square test, Fisher's exact test, variance test, nonparametric Wilcoxon test, and Spearman correlation test.

## 3. Results

The overall success rate was 71.4% (95% confidence interval), meaning that 15 patients (out of 21) had no adverse event and no reoperation during the whole follow-up. Reoperation rate was low (4.8%, *N* = 1) and neither negative effects linked to the use of PEEK-OPTIMA rods nor rod fracture or displacement has been reported. A total of 6 complications were reported. Among these complications only 1 (an irritation of a nerve root caused by a pedicle screw) required an additional surgical procedure to replace the screw. Four patients expressed some minor remaining pains, which did not affect their satisfaction with the operation. Another patient noted on the questionnaire a neurological deficit in the lower left limb, this being already present prior to the operation.

The results of quality of life, evaluated using VAS and ODI, are summarised in Table 1. VAS scores did not exceed 2.7 when moving. The mean ODI was 16.0% (std. 15.9%), with a median of 12.0% (IQR 2–22). The average ODI was 21.6 (std. 22.1) for smokers and 15.6 (std. 14.4) for nonsmokers (*p* = 0.49), which categorises all the patients as having “moderate disability,” with a clear advantage for the nonsmokers.

The mean PSI (patient satisfaction index evaluated on a scale of (1) to (4), (1) being the best result) was 1.4 (std. 0.9) with a median of 1 (IQR 1-1), which therefore means that all the patients were satisfied with their operations.

The radiological outcomes regarding progression of adjacent discs are shown in Table 2. There was nearly 80% preservation of the original disc height at the upper adjacent level and over 70% at the lower adjacent level, whether a disc is initially healthy or partially degenerated.

**Figure 2:**
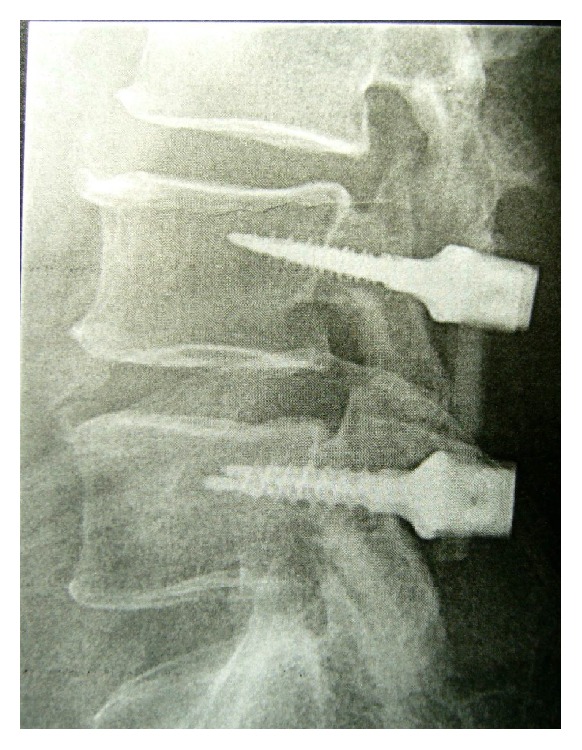
Lateral radiograph of the lumbar spine of a 74-yo patient with a canal stenosis shows spinal stability plus segmental and adjacent discs preservation.

**Figure 3:**
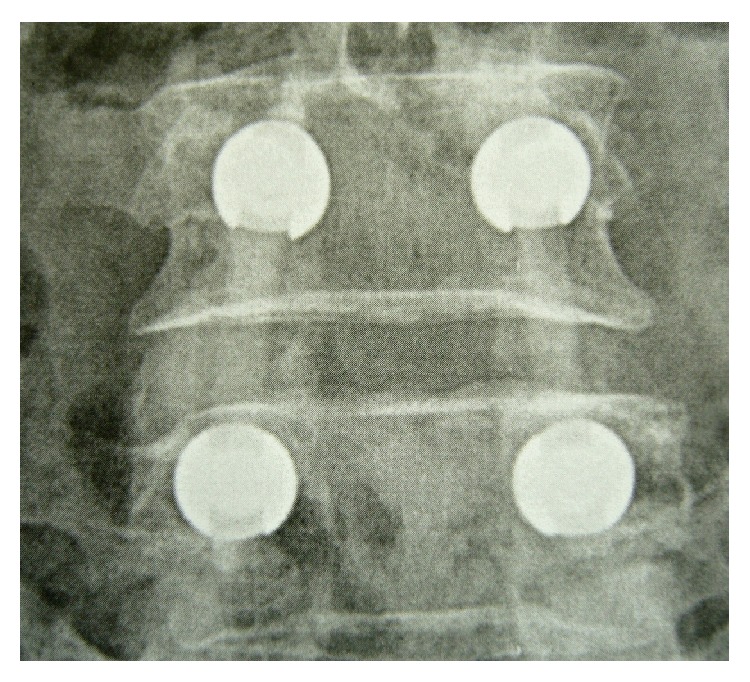
AP view of the same patient. The good radio lucency of PEEK rods enhances postoperative assessment.

**Figure 4:**
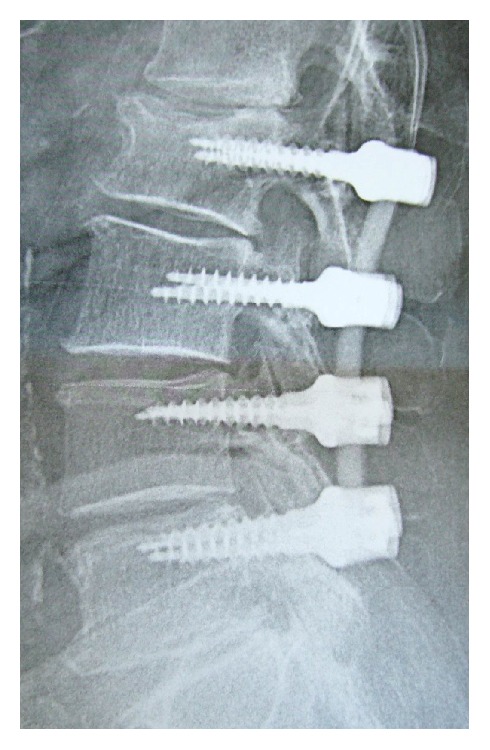
Lateral view of a 71-yo patient of PEEK rods stabilisation at 4 levels without fusion. Sagittal balance and disc height are preserved.

**Figure 5:**
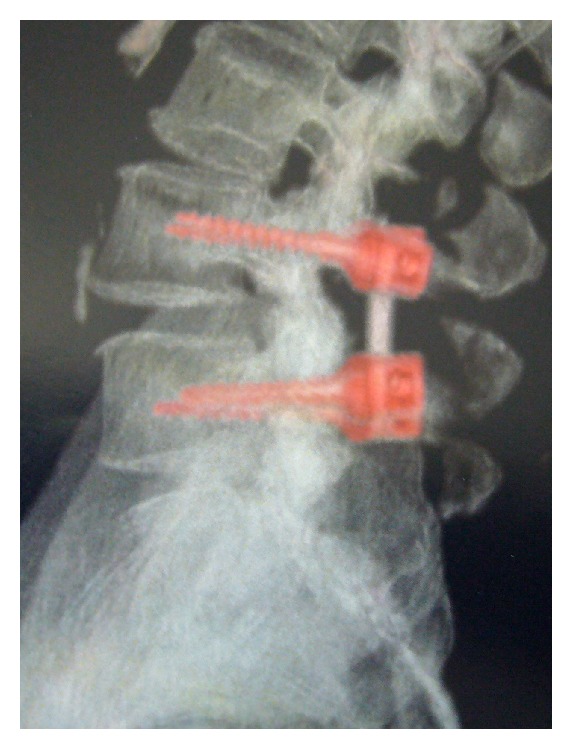
CT scan of another 71-yo patient with bone window shows clearly the PEEK rod and can detect a possible rupture.

PEEK's good X-ray compatibility enabled high-quality postoperative analysis with few artefacts in constructs on one level (Figures 2 and 3) or in constructs on several levels (Figure 4) that fully maintained lumbar sagittal balance. Bone window tomodensitometric analysis provided perfect visibility and clarity of the rods themselves whose integrity is easy to confirm or otherwise (Figure 5).

Finally, relation between the number of surgical levels, the appearance of upper and lower adjacent secondary degeneration, and the existence of complications was investigated, but no correlation could be demonstrated.

## 4. Discussion

The objectives of this retrospective clinical study were to validate this technique by demonstrating the values of PEEK-OPTIMA rods systems compared to rigid ones when looking at complication and reoperation rates, patients' quality of life and satisfaction, and evolution of the adjacent discs. Given the results presented above, the complication and reoperation rates (4.8%, *N* = 1) and patients' quality of life (ODI = 16%) and satisfaction (PSI = 1.4) are as good as or even better than the ones reported in the literature [27, 28]. PEEK rods provide a secured flexible stabilisation with a low rate of both reoperations and serious complications (4.8%) and the absence of negative effects linked to the use of PEEK-OPTIMA rods, no fracture, and no displacement. Moreover, the high level of preservation of adjacent upper disc (75%) can predict a long-term quality of stabilisation.

The main limitation of this paper is the short follow-up period, although one of the longest of those published in the literature, and all patients were operated on by the same surgeon. Nevertheless, the present results are consistent with the previous studies. It has actually been demonstrated that the PEEK rods constructs provide the same immediate stability than a rigid construct, in terms of reduction of range of motion (angular displacement) [29, 30]. In terms of fatigue resistance, Chou et al. [31] demonstrate that stability is maintained with PEEK rods constructs after fatigue testing (90,000 flexion/extension cycles), whereas there is a loss of stability with titanium (rigid) system. The authors highlight the reduction of stress (at the rod and at the bone/screw interface) and the protection of adjacent level with PEEK rods. Therefore the preconception of rod failure because of the flexibility of PEEK is not supported and, on the contrary, the risk of failure (breakage and loosening) is higher with rigid systems [32].

“Load sharing” is also another concept to discuss. It is commonly accepted that the anterior corporeo-discal segment of the spinal column supports 80% of the applied stresses, leaving only 20% of these stresses on the posterior articular segment [33]. During the degenerative process, there is a load transfer onto the posterior segment which would explain the source of the lower back pains observed in this disease [34]. Rigid titanium rods impose considerable posterior stresses by unloading compression forces from the anterior segment and put the bone-screw interface at a high level of tension, increasing the risks of avulsion or fracture of the construct at that level [29, 34, 35]. Due to their reduced rigidity, PEEK rods simulate a physiological situation that is closer to what is normal in terms of load sharing between the anterior and posterior vertebral compartments, which decreases the chances of the degenerative disease occurring as well as the risks of fracture of the pedicle or of bone avulsion from the device, through less stress on the bone-screw interface [36]. These are the concepts of “better load sharing” and “less bone-screw interface stresses” developed by Turner et al. [37].

The number of lumbar arthrodeses performed for the purpose of stabilising the lumbar spine has increased considerably during the last ten years. In the United States, where all surgical procedures are systematically reported, there was a 220% increase in the number of spinal fusion operations between 1990 and 2001 [38, 39]. Alongside this increase, over the 4 years there was a growth of approximately 15% [8] in the number of reoperations, with a 40% chance of reoperation within the following year for patients who had undergone an arthrodesis, the majority (65%) of these repeat procedures during the year being due to the materials used. PEEK rods could be proposed as a solution (in order to decrease the complication rate and protect adjacent levels) since these degenerative problems arise in an ageing population (average age in our case series was 70 years) weakened by the existence of comorbidity and often presenting problems of osteoporosis that further increase the risks of immediate or secondary osteosynthetic failure. By performing a stabilisation only with PEEK rods (no cage or other type of devices implanted), the operating time is considerably shortened, the immediate stability is achieved, and the complications associated with positioning cages are avoided. Also, the likelihood of disease occurring in one or more of the adjacent segments is diminished by maintaining a more physiological vertebral mechanism, suitable for those degenerative spines.

How PEEK rods systems differentiate themselves from pure dynamic ones [33] can be also discussed: among them, Graf ligament [40, 41], Scient'x Isolock [42], and Zimmer Dynesys [43–45] are the best known. The purpose of these various systems is to maintain motion in the disc space in the manner of a disc prosthesis while ensuring stability in this area. Compared to these systems (especially Isolock), we have found the implantation of PEEK rods more straightforward and intuitive (short learning curve and no risk of loss of biomechanical dynamic properties by inadequate prestressed position in vivo). We could also mention here the various interspinous systems that cannot claim an equivalent stabilisation of the disc space involved, in the biomechanical sense of the term [46, 47] compared to PEEK rods.

Other authors prior to ourselves have proposed the use of nonrigid material to stabilise the lumbar spine but almost always associated with an arthrodesis, the aim being to increase the chances of fusion by making use of the mechanical properties of PEEK type materials that provide improved load sharing on the vertebral body and thus, in accordance with Wolff's law, an earlier and higher quality fusion. In a comparative study of 60 patients treated with PEEK rods and 60 patients treated with titanium rods, Pasciak et al. [48] find improved long-term results with PEEK rods but the follow-up periods remain short (less than 2 years). Galler [49], in a retrospective study of 30 cases, finds no fracture in the material after 1 year and concludes that PEEK rods are an alternative to titanium rods. In Ormond et al.'s [50] case series of 42 patients, PEEK rods were not found to be inferior to those made of titanium but neither were there advantages. De Iure et al. [51] report on a retrospective study of 40 cases with an 18-month follow-up and conclude that the use of PEEK rods for degenerative lumbar disease may be considered as a future option. In all these case series, PEEK rods were used for arthrodesis purposes. A recent study (2013) published by Qi et al. [52] confirms the equivalent performance of PEEK rods when compared with titanium, but the author considers the technique's high costs to be a handicap. Athanasakopoulos et al. [28] report a series of 52 patients who underwent posterior spinal fusion using PEEK rod from 2007 and 2010. ODI is around 28% and 1 patient required a new surgery; no adjacent segment degeneration was observed. The authors also conclude that PEEK rods systems provide excellent early clinical results. Mavrogenis et al. [27] present a general review of the clinical use of PEEK rods where the previously described advantages are repeated. Only Highsmith et al. [53], out of 3 cases, report on an 80-year-old patient simply stabilised with PEEK rods for a severe lumbar stenosis with pseudospondylolisthesis. They conclude that PEEK rods are a good intermediate option between totally dynamic systems such as prostheses or Dynesys and rigid fixation systems with titanium rods and screws.

## 5. Conclusion

In conclusion, looking at the biomechanical and clinical data already published and based on the results of our series, PEEK-OPTIMA spinal rods are a safe and effective alternative solution to rigid systems leading to low rate of complications and revisions, high rate of adjacent disc preservation, high degree of patient's quality of life, and satisfaction. PEEK-OPTIMA can be considered a suitable material for rods to stabilise a degenerative lumbar column without additional risk for the patients, particularly if it is used as a means of stabilisation without arthrodesis. PEEK-OPTIMA's mechanical characteristics, especially its reduced rigidity and high fatigue resistance, provide the appropriate load sharing on the lumbar column to create more favourable conditions for the adjacent discs and reduce the likelihood of the appearance of secondary deterioration and therefore a further operation.

## Figures and Tables

**Table 1:** Results of quality of life, evaluated using VAS and ODI.

Visual Analog Scale (VAS)		Oswestry Disability Index (ODI)	
Characteristics	Mean [95% CI]	ODI classes	*N* (%)
Pain when moving	2.7 [1.4–4.1]	0–4: no incapacity	7 (33.3)
Pain when standing up	2.2 [1.3–4.0]	5–14: light incapacity	5 (23.8)
Pain when sitting	1.5 [0.5–2.5]	15–24: moderate incapacity	4 (19.1)
		25–34: severe incapacity	2 (9.5)
		>34: complete incapacity	3 (14.3)

**Table 2:** Degenerative status of adjacent discs (above and below) at the final evaluation and compared to the initial status.

Characteristics	Lower level *N* (%)	Upper level *N* (%)
Degeneration on last evaluation		
None	14 (73.7)	15 (79.0)
Doubtful	0	0
Minimal	3 (15.8)	3 (15.8)
Moderate	1 (5.3)	0
Severe	1 (5.3)	1 (5.3)
*Missing*	*2*	*2*
Evolution		
Absent initially and from last evaluation	12 (70.6)	13 (68.4)
Present initially and in last evaluation	5 (29.4)	2 (10.5)
Absent initially and present in last evaluation	0	2 (10.5)
Present initially and absent from last evaluation	0	2 (10.5)
*Missing*	*4*	*2*
